# Correction to “Qualitative Analysis of Blood From Patients Engaging in Deliberate Self‐Harm: Differences Between Prescribed and Detected Drugs”

**DOI:** 10.1002/npr2.12513

**Published:** 2024-12-25

M. Masuda, B. Waters, L. Gotoh, Y. Nakamura, Y. Kato, S. Nabeshima, et al. “Qualitative Analysis of Blood From Patients Engaging in Deliberate Self‐Harm: Differences Between Prescribed and Detected Drugs.” *Neuropsychopharmacology Reports* 44 (2024): 809–820.

In Table 1, the “Violent Methods” category has missing symbols. Below is the updated Table 1:

**TABLE 1:** Summary of all DSH cases of the study (*n* = 55).

	Male	Female	*p*
	20 (36.0%)	35 (64.0%)	
Age (years)	46.5 ± 16.4	24.9 ± 9.4	< 0.0001*
History of psychiatric treatment			0.041*
Yes	13 (65.0%)	32 (91.4%)	
No	6 (30.0%)	3 (8.6%)	
Unknown	1 (5.0%)	0 (0%)	
Diagnosis (ICD‐10)			0.257
F1 (Psychoactive substance use)	4 (20.0%)	3 (8.6%)	
F2 (Schizophrenia)	5 (25.0%)	2 (5.7%)	
F3 (Mood disorders)	5 (25.0%)	11 (31.4%)	
F4 (Neurotic disorders)	1 (5.0%)	5 (14.3%)	
F6 (Personality disorders)	0 (0%)	2 (5.7%)	
F7 (Mental retardation)	0 (0%)	1 (2.9%)	
F8 (Developmental disorders)	2 (10.0%)	7 (20.0%)	
F9 (Behavioral and emotional disorders)	1 (5.0%)	3 (8.6%)	
Unknown	2 (10.0%)	1 (2.9%)	
History of DSH			0.004*
Yes	9 (45.0%)	30 (85.7%)	
No	9 (45.0%)	5 (14.3%)	
Unknown	2 (10.0%)	0 (0%)	
Family history of psychiatric treatment			0.401
Yes	3 (15.0%)	6 (17.1%)	
No	16 (80.0%)	29 (82.9%)	
Unknown	1 (5.0%)	0 (0%)	
Method of recent DSH			0.080
Overdose (Medications/OTC)	8 (40.0%)	20 (57.1%)	
Poisoning (Other substances)	4 (20.0%)	1 (2.9%)	
Jumping	4 (20.0%)	11 (31.4%)	
Stabbing/Cutting	2 (10.0%)	2 (5.7%)	
Hanging	2 (10.0%)	0 (0%)	
Self‐Immolation	0 (0%)	1 (2.9%)	
Violent methods			1.000
Yes (Violent)^a^	8 (40.0%)	14 (40.0%)	
No (Non‐violent)^b^	12 (60.0%)	21 (60.0%)	
Alcohol‐use			0.333
Yes	3 (15.0%)	10 (28.6%)	
No	17 (85.0%)	25 (71.4%)	

Abbreviations: DSH, deliberate self‐harm; ICD, International Classification of Diseases; OTC, over‐the‐counter drugs.

^a^ Includes jumping, stabbing/cutting, hanging, and self‐immolation.

^b^ Includes overdose of medications/OTC or poisoning by other substances.

* *p* < 0.05.

We apologize for this error.

